# From anthropomorphism to prosocial intention: a PLS-SEM and fsQCA study of virtual influencers

**DOI:** 10.3389/fpsyg.2026.1899105

**Published:** 2026-07-28

**Authors:** Lan Su, Xiaolong Pan, Xinyao Duan

**Affiliations:** 1 School of Fine Arts and Design, Quanzhou Normal University, Quanzhou, China; 2 School of Business, Monash University Malaysia, Subang Jaya, Malaysia; 3 Institute for Advanced Studies, Universiti Malaya, Kuala Lumpur, Malaysia

**Keywords:** anthropomorphism, environmental self-identity, parasocial interaction, prosocial intention, virtual influencers

## Abstract

**Introduction:**

Virtual influencers are increasingly deployed to mobilize prosocial causes, yet the mechanisms converting humanlike design into action remain under-specified. This study integrates social cognitive theory with identity perspectives to examine how anthropomorphism fosters parasocial interaction and, in turn, attitudes, environmental self-identity, and prosocial intention.

**Methods:**

Using purposive sampling, data were collected from 209 individuals familiar with virtual activist content. The proposed model was assessed using partial least squares structural equation modeling, and the net-effect findings were complemented with fuzzy-set qualitative comparative analysis.

**Results:**

Anthropomorphism significantly increased parasocial interaction and attitudes toward the influencer. Parasocial interaction positively predicted attitudes, environmental self-identity, and prosocial intention. Attitudes and environmental self-identity directly predicted intention and mediated the effects of parasocial interaction. The fsQCA results identified multiple configurations associated with high intention, centered on environmental self-identity, and revealed causal asymmetry in the configurations associated with low intention.

**Discussion:**

The findings formalize activist anthropomorphism as a higher-order construct and demonstrate parallel evaluative and identity-based pathways to prosocial intention. The study extends virtual influencer research beyond commercial persuasion and offers guidance for designing credible and humanlike virtual advocates for environmental causes.

## 1 Introduction

Virtual influencers have evolved from digital curiosities into strategically managed communicators who shape engagement and persuasion in both commercial and cause oriented campaigns (De Cicco et al., 2024; Lou et al., 2022; Sorosrungruang et al., 2024). Recent studies show that content produced by computer generated imagery influencers can elicit meaningful user engagement, particularly when emotional displays are salient and humanlike cues are present, suggesting that designed social signals can substitute for human presence in driving responses online (Yu et al., 2024). Comparative evidence further indicates that virtual and human influencers can generate similar downstream effects on perceived credibility, competence, and brand attitudes (De Cicco et al., 2024; Yoo et al., 2024). These developments sharpen an important question for prosocial communication: when activist messages are delivered by artificial agents, which

psychological mechanisms translate engineered human likeness into intentions to act for social and environmental good.

Foundational work on anthropomorphism explains why individuals respond socially to non-human agents. Epley et al. (2007) propose that people attribute humanlike minds based on accessible agent knowledge, effectance motivation, and sociality motivation, while subsequent research shows that even minimal cues in digital interfaces can trigger social responses that mirror interpersonal treatment (Nass and Moon, 2000; Seeger et al., 2021). In parallel, parasocial interaction research conceptualizes the one-sided sense of intimacy and reciprocity that arises through repeated exposure to media figures, a mechanism now central to understanding how influencers mobilize audiences on social platforms (Horton and Wohl, 1956; Tukachinsky et al., 2020). Emerging evidence suggests that users also form parasocial bonds with virtual influencers and that perceived human likeness strengthens these bonds and shapes source evaluations (Liu, 2023; Stein et al., 2022). At the same time, research on environmental self-identity demonstrates that seeing oneself as a pro environmental person is a proximal predictor of preferences, intentions, and behaviors (van der Werff et al., 2013; Brick and Lai, 2018; Ajibade and Boateng, 2021). Identity therefore offers a theoretically distinct pathway through which mediated encounters with activist figures may translate into prosocial action.

Despite this growing body of work, several gaps remain. First, much virtual influencer research has been conducted in commercial contexts and has focused on outcomes such as purchase intention, engagement, and brand attitudes rather than prosocial or environmental intentions (Casaló et al., 2020; Filieri et al., 2022; Han and Balabanis, 2023). Studies on greenfluencers and sustainability communication show that influencers can function as social change agents (Kapoor et al., 2023), yet the literature has only begun to examine how virtual influencers might mobilize prosocial behavior in similar ways (Igarashi et al., 2024). Second, although anthropomorphism has been extensively discussed, recent work highlights increasingly fine-grained conceptualizations and effects in automated and AI-enabled agents (Dabiran et al., 2024; Geiselmann et al., 2023; Yin et al., 2026). However, the mechanisms through which multidimensional anthropomorphic design features in virtual influencers operate through organismic states such as parasocial interaction, attitudes, and environmental self-identity to shape prosocial intentions remain under specified. Third, most extant models treat attitudes and identity in isolation rather than as potentially parallel channels from the same upstream social cognitive processes, even though theory implies that both relational bonding with a messenger and alignment with a valued self-concept can energize prosocial intentions (Bandura, 1986, 2001; Hoffner and Bond, 2022; Udall et al., 2021). Finally, research on virtual agents increasingly acknowledges concerns related to the uncanny valley, where excessive realism may diminish positive responses (Mori et al., 2012; Stein et al., 2022), yet empirical models rarely consider how different levels or facets of anthropomorphism might differentially support prosocial persuasion in activist contexts.

The objective of this study is to examine how multidimensional anthropomorphic cues in virtual activist influencers shape prosocial environmental intention through parasocial interaction, attitude toward the influencer, and environmental self-identity. The present study addresses these gaps by developing and testing a dual process model of virtual influencer activism that integrates anthropomorphism, parasocial interaction, attitudes toward the influencer, environmental self-identity, and intention to engage in prosocial behaviors. Drawing on social cognitive theory, anthropomorphism is conceptualized as a higher-order stimulus comprising four validated facets: appearance, moral virtue, cognitive experience, and conscious emotional qualities that enhance model salience and social presence (Bandura, 2001; Golossenko et al., 2020; Yoo et al., 2024). These design features are theorized to strengthen parasocial interaction with the virtual activist, which in turn influences two organismic states: evaluative responses in the form of attitude toward the influencer and identity-based responses in the form of environmental self-identity (Chung and Cho, 2017; Joyce and Harwood, 2014; van der Werff et al., 2013). The model thus specifies parallel evaluative and identity pathways from anthropomorphism to prosocial intention, rather than privileging a single route.

The study advances theory and practices in several ways. Conceptually, it formalizes activist anthropomorphism as a multidimensional, higher order construct in the context of virtual influencers and clarifies how it operates through parasocial bonding to shape both attitudes and environmental self-identity. This responds directly to calls to move beyond generic anthropomorphism measures and to integrate identity-based mechanisms into models of prosocial behavior in mediated settings (Brick and Lai, 2018; Udall et al., 2021; Wang, 2024). Empirically, the study focuses on environmental prosocial intention, thereby extending virtual influencer research from commercial outcomes to sustainability relevant behavior. Methodologically, it combines partial least squares structural equation modeling with fuzzy set qualitative comparative analysis to examine both net effects and multiple sufficient configurations, including the role of environmental self-identity as a potential necessary condition for high prosocial intention (Fang et al., 2016; Fiss, 2011; Pappas and Woodside, 2021).

## 2 Literature review

### 2.1 Social cognitive theory

Social cognitive theory (SCT) provides an agentic account of how symbolic communication alters cognition, affect, and behavior through observational learning, vicarious reinforcement, and self-regulatory processes operating within triadic reciprocal causation among person, behavior, and environment (Bandura, 2001). Observational learning directs attention to salient models, encodes their scripts and outcome contingencies, and motivates enactment via vicarious reinforcement and self-evaluative standards. In mass communication, repeated exposure to compelling models establishes performance heuristics and expectancies while activating self-regulatory mechanisms that guide future choice without requiring direct interpersonal contact (Zimmerman, 2013). These mechanisms render SCT particularly apt for influencer-based persuasion, where audiences learn from parasocially encountered figures who model norms, display consequences, and invite audience comparison with internal standards (Bandura, 1986).

Applied to the present model, anthropomorphism functions as a design lever that heightens model salience and social presence (Yoo et al., 2024), thereby increasing attention and perceived relevance of the virtual activist, which facilitates parasocial interaction as the experiential correlate of observational engagement (Horton and Wohl, 1956; Stein et al., 2022). SCT then accounts for two complementary organismic outputs of this engagement (Bandura, 1986). First, evaluative alignment arises as audiences internalize the model's stances and outcome expectancies, strengthening attitudes toward the influencer as a proximal determinant of intention (Chung and Cho, 2017; Filieri et al., 2022). Second, self-alignment follows from SCT's self-reflective and self-regulatory subfunctions: exposure to modeled pro-environmental norms prompts self-appraisal and calibration of standards, reinforcing environmental self-identity as a moral self-belief that energizes action (van der Werff et al., 2013; Hoffner and Bond, 2022). Parasocial interaction and identification are distinct but related processes within SCT that support both outputs (Gleason et al., 2017; Joyce and Harwood, 2014), with attitudes and identity operating as parallel channels from the same learning episode. A critical implication is that environmental structures also matter. Platform affordances and algorithmic curation shape exposure patterns and reinforcement histories (Bucher, 2018), thus constituting the environmental arm of triadic causation that conditions how anthropomorphic cues and parasocial processes translate into intention (Bandura, 2001).

### 2.2 Virtual influencers and virtual activism

Virtual influencers are computer-generated social media personas that communicate with online audiences in ways similar to human influencers. They are typically designed with names, appearances, personalities, lifestyles, and narrative identities, allowing them to endorse products, interact with followers, and participate in social or cultural conversations (Lou et al., 2022; Stein et al., 2022; Sorosrungruang et al., 2024). Unlike human influencers, however, virtual influencers are fully or partly constructed by creators, agencies, or brands. This makes them highly controllable and scalable, but it also raises questions about authenticity, transparency, credibility, and moral agency (Arsenyan and Mirowska, 2021; Robinson, 2020).

In recent years, virtual influencers have moved beyond fashion, entertainment, and commercial endorsement into cause-related communication and virtual activism. Virtual activism refers to the use of digital platforms, mediated personas, and online narratives to advocate for social, ethical, or environmental causes. In this context, virtual influencers may post about sustainability, ethical consumption, climate awareness, animal welfare, recycling, or other prosocial issues. For example, Lil Miquela has been widely discussed as one of the most visible humanlike virtual influencers and has been associated with social and cause-related messaging (Neri, 2022). Noonoouri is frequently positioned in relation to veganism, anti-fur messaging, and sustainable fashion, while Leya has been examined as a virtual influencer using nature-centered visuals and storytelling to promote environmental awareness (Tung, 2024).

### 2.3 Intention to engage in prosocial behavior

Intention to engage in prosocial behavior refers to the willingness of individuals to engage in behaviors that contribute to societal benefits, including donating and volunteering (Conde and Casais, 2023). Intention has been well explained through cognitive and motivational models (Ajzen, 1991) and extended frameworks incorporating self-identity (van der Werff et al., 2013). This suggests that identity may be relevant in the mediated contexts where individuals align with symbolic figures. For example, Kapoor et al. (2023) found that greenfluencers have the power as social change agents in influencing consumers to purchase sustainable products.

The effectiveness of virtual influencers in stimulating consumer behavior has been seen as equally effective as human influencers in eliciting prosocial intentions when the credibility of the virtual influencers is successfully engineered (Igarashi et al., 2024). However, the authors also point out that there are limited studies on the effectiveness of how virtual influencers promote prosocial causes and their impact on the followers' prosocial intention.

Studies have shown that influencers with high parasocial interaction increase compliance with recommendations and prosocial actions such as donations and gifting (Conde and Casais, 2023; Liu et al., 2025). Given that virtual influencers can be anthropomorphised to evoke perceptions, feelings of social presence, and realign followers' self-identity, it is believed that virtual influencers foster supportive behavior among the followers (Lin, 2021). While most studies have focused on human influencers, we believe that virtual influencers can also deliver similar persuasion in promoting prosocial intention among followers.

### 2.4 Anthropomorphism in virtual influencer

Virtual influencers are purposefully designed, computer generated personas that communicate on social platforms on behalf of brands and causes (Lou et al., 2022; Sorosrungruang et al., 2024). Their perceived effectiveness rests in part on the extent to which audiences experience them as humanlike communicators. Anthropomorphism provides a foundational explanation for this response. According to Epley et al. (2007), people attribute humanlike minds to non-human agents when agent knowledge is accessible and when they are motivated by effectance and sociality. Consistent with the Computers are Social Actors paradigm, even minimal social cues in interfaces can elicit social treatment that resembles responses to human partners (Nass and Moon, 2000). More recent work extends these insights to automated and AI enabled agents, showing that carefully calibrated anthropomorphic cues can enhance trust, engagement, and perceived credibility (Seeger et al., 2021; Dabiran et al., 2024; Yin et al., 2026).

Mind perception research further refines anthropomorphism by distinguishing between perceptions of agency and experience and by demonstrating that these dimensions shape moral judgments and social responses (Gray et al., 2007; Haggard and Tsakiris, 2009; Geiselmann et al., 2023). For virtual influencers, cues that signal cognitive capacity and affective experience are therefore likely to be particularly influential in driving downstream relational and evaluative outcomes. Building on this work, Golossenko et al. (2020) conceptualize anthropomorphism as a multidimensional construct comprising appearance related cues, perceived moral qualities, cognitive experience, and conscious emotional capacities. These four facets reflect the breadth of humanlike signals available to designers and offer a structured basis for measuring how audiences perceive virtual agents as humanlike.

The present study adopts this multidimensional perspective and operationalises anthropomorphism as a higher order construct formed by four lower order dimensions. Appearance captures the extent to which the influencer visually resembles a human in form and expression. Moral virtue reflects perceived integrity and ethical standing of the influencer as a social actor. Cognitive experience refers to perceived capacity for thinking, reasoning, and understanding, while conscious emotional qualities refer to perceived ability to feel and express emotions in a way that appears subjectively real to observers (Golossenko et al., 2020). Together, these facets capture both surface level and deeper mind related aspects of human likeness. Their joint consideration is especially relevant in activist contexts, where followers must not only find the virtual influencer visually acceptable but also perceive the agent as morally trustworthy, cognitively competent, and emotionally engaged with the cause.

Empirical work in virtual influencer research supports these expectations. Studies show that increased human likeness is associated with stronger parasocial responses, higher credibility, and more favorable source evaluations, if authenticity concerns are managed effectively (Stein et al., 2022; De Cicco et al., 2024; Yoo et al., 2024). Recent investigations of AI driven influencers indicate that anthropomorphic design enhances persuasive impact when users perceive the agent as morally grounded and cognitively capable, rather than relying on visual realism alone. Evidence shows that attributes related to sincerity, ethical character, and thoughtful expression strengthen credibility and deepen parasocial relationships, both of which are critical pathways to influencing consumer intentions, whereas appearance-based anthropomorphism contributes only weakly to persuasive outcomes (Dabiran et al., 2024; Yin et al., 2026). In prosocial and sustainability-oriented communication, such features are likely to be especially important because they facilitate relational bonding and perceived alignment between the influencer's stance and the follower's emerging self-concept as an environmentally responsible person (Kapoor et al., 2023; Wang, 2024). Hence, we propose the following hypothesis:

H1: Anthropomorphism positively predicts parasocial interaction.

H2: Anthropomorphism positively predicts attitude toward the influencer.

### 2.5 Parasocial interaction as the social cognitive conduit

Parasocial interaction refers to the one-sided sense of intimacy and reciprocity that audiences experience with media figures during exposure (Horton and Wohl, 1956). The construct was introduced to explain how conversational cues and repeated contact produce feelings that mimic interpersonal exchange, even in the absence of true mutuality, and this mechanism has since been observed across traditional and social media contexts. Contemporary work specifies cognitive, affective, and conative components of parasocial responding. It shows that such bonds reliably develop with social media influencers, including virtual agents, when design cues invite engagement (Stein et al., 2022).

A substantial body of evidence links parasocial interaction to downstream evaluations and conative outcomes on platforms where influencers operate. Empirical studies document that stronger parasocial bonds enhance attitudes toward the messenger and strengthen intentions in domains ranging from product choice to continued engagement (Chung and Cho, 2017). Meta-analytic reviews show robust positive associations between parasocial ties and behavior (Tukachinsky et al., 2020). These patterns justify treating parasocial interaction as a proximal social cognitive mechanism that channels upstream cues into evaluative judgments and behaviourally relevant motivation.

In the specific case of virtual influencers, experiments comparing human and virtual sources report that audiences do form parasocial reactions toward virtual personas of comparable design quality, which in turn predict message evaluations (Stein et al., 2022). Studies in adjacent interactive contexts, such as live streaming and charity streaming, further show that perceived viewer-streamer interaction rooted in parasocial dynamics is associated with social support and gifting intentions, offering convergent evidence that parasocial processes translate mediated rapport into action tendencies (Wang, 2024).

Beyond attitudes and intentions, parasocial interaction can shape identity-relevant inferences. Joyce and Harwood (2014) found that identification with modeled others and vicarious processes can inform the self, especially when exposure is repeated and norms are salient. Developmental and media research similarly indicates that parasocial ties participate in identity work, suggesting a plausible route from parasocial interaction to stronger self-diagnostic beliefs aligned with a communicator's cause (Gleason et al., 2017). When the cause is environmental, such identity-relevant inferences are theoretically compatible with shifts in environmental self-identity, which is known to predict prosocial action.

Prior research shows that parasocial relationships increase followers' receptiveness to influencer recommendations and strengthen behavioral intentions across social media contexts (Chung and Cho, 2017; Sokolova and Kefi, 2020; Tukachinsky et al., 2020). In prosocial and cause-related settings, parasocial bonds have been linked to greater social support, donation intention, gifting intention, and environmental participation (Conde and Casais, 2023; Dekoninck and Schmuck, 2024; Lin, 2021; Liu et al., 2025; Wang, 2024). These findings suggest that when followers feel a sense of closeness and familiarity with a virtual influencer, they may be more willing to act in line with the influencer's advocated cause. Hence, we propose the following hypothesis:

**H3:** Parasocial interaction positively predicts attitude toward the influencer.

**H4:** Parasocial interaction positively predicts environmental self-identity.

**H5:** Parasocial interaction positively predicts intention to engage in prosocial behaviors.

### 2.6 Attitude toward the influencer

Attitude toward the influencer is a global evaluative judgment of the messenger that consolidates perceptions of attractiveness, expertise, and trustworthiness into an overall valence guiding compliance (Filieri et al., 2022). Research on social media influencers consistently links favorable attitudes toward the source to stronger behavioral intentions, both directly and as part of broader persuasion processes (Belanche, 2021; Sánchez-Fernández and Jiménez-Castillo, 2021). For example, evidence from large samples of Instagram followers shows that influencer attributes shaping opinion leadership translate into downstream intentions to follow advice and recommend content, implying that positive source evaluations are a proximal lever of conation (Casaló et al., 2020). Meta-analytic syntheses further indicate that influencer effects on engagement and purchase intention operate in part through source-related evaluations such as credibility and attractiveness, which are tightly coupled with overall attitude toward the influencer (Han and Balabanis, 2023). Virtual influencer research similarly shows that perceived attractiveness, when evaluated positively, enhances users' appraisal of the source and indirectly influences intention outcomes through mechanisms such as mimetic desire and brand attachment, highlighting the generality of this evaluative pathway across both human and synthetic endorsers (Kim and Park, 2023).

Parasocial interaction strengthens attitude toward the influencer by rendering the messenger more familiar, intimate, and socially present. Studies of celebrity and social media endorsement show that parasocial bonds developed through ongoing social media contact and self-disclosure cues elevate perceptions of source trustworthiness, which enhances brand credibility and predicts stronger purchase intentions (Chung and Cho, 2017). In influencer contexts specifically, parasocial interaction and source evaluations jointly predict intention, with parasocial ties enhancing the favourability of the influencer and thereby facilitating acceptance of advocated recommendations (Sokolova and Kefi, 2020). It indicates that when audiences experience a sense of one-sided intimacy with an activist influencer, they are more likely to form favorable attitudes toward the messenger and translate those evaluations into intentions to engage in advocated behaviors.

Taken together, the literature positions attitude toward the influencer as a distinct evaluative channel that complements identity-based processes. Parasocial interaction functions as an upstream relational mechanism that enhances attitudes toward the influencer. These attitudes exert a direct effect on intention. Therefore, beyond any direct parasocial effect on intention, a mediated route via attitude is theoretically warranted. Hence, we proposed the following hypothesis:

**H6:** Attitude toward the influencer positively predicts intention to engage in prosocial behaviors.

**H8:** Attitude toward the influencer mediates the relationship between parasocial interaction and intention to engage in prosocial behaviors.

### 2.7 Environmental self-identity

Environmental self-identity captures the extent to which people view themselves as the kind of person who acts in environmentally responsible ways (van der Werff et al., 2013). A large body of evidence shows that this identity is a proximal predictor of environmental preferences, intentions, and behaviors (Brick and Lai, 2018; van der Werff et al., 2013). In multi-study evidence, environmental self-identity mediates the influence of biospheric values on pro-environmental intentions and behavior, indicating that values become action-guiding when they are linked to the self (Jia and Liang, 2024; van der Werff et al., 2021; Whitmarsh and O'Neill, 2010).

Furthermore, environmental self-identity is also responsive to signals and experiences that affirm one's role as a pro-environmental actor (Udall et al., 2021). Past pro-environmental choices can strengthen identity by signaling to the self that one is the kind of person who cares for the environment, which in turn predicts subsequent intentions and behavior (Carfora et al., 2017; van der Werff et al., 2014). This dynamic underscores identity's motivational function in sustaining goal-consistent action.

Additionally, parasocial processes offer a theoretically grounded route through which identity-relevant inferences can be shaped in mediated settings (Wang, 2024). SCT explains that repeated exposure to modeled others and vicarious reinforcement informs self-views and scripts for action (Bandura, 1986). Media studies on identification and parasocial relationships similarly indicate that close psychological connections with media figures can contribute to identity work (Hoffner and Bond, 2022). In influencer contexts, parasocial bonds personalize the messenger and render the advocated role more self-congruent (Gleason et al., 2017). It makes it plausible that stronger parasocial interaction elevates environmental self-identity in activist campaigns. Hence, we proposed the following hypothesis:

**H7:** Environmental self-identity positively predicts intention to engage in prosocial behaviors.

**H9:** Environmental self-identity mediates the relationship between parasocial interaction and intention to engage in prosocial behaviors.

**Figure 1:**
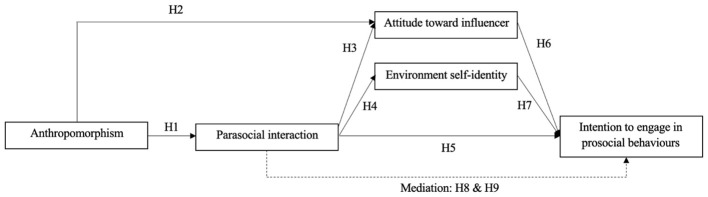
Conceptual framework.

Based on the nine hypotheses, the conceptual framework is shown in Figure 1.

## 3 Methodology

### 3.1 Participants and procedure

This study employed purposive sampling to recruit individuals familiar with virtual influencer activism. Data collection was conducted through Prolific after the study was published on 12 December 2024, an online research recruitment platform that allows researchers to invite participants from a pre-screened participant pool based on study eligibility criteria (Eyal et al., 2021). On Prolific, the study was listed as a survey and participants were directed to an external Google Form to complete the questionnaire. The questionnaire was administered in English. Participants were recruited through Prolific from multiple national contexts rather than from a single country, as reflected in the sample distribution reported in Table 1. Participants received financial compensation through Prolific in accordance with the platform's payment procedure. The study description informed potential participants that the research examined virtual influencers and prosocial environmental behavior, and specifically invited individuals familiar with virtual influencers, such as Lil Miquela, to participate. No formal pilot test was conducted prior to full data collection. However, the questionnaire was developed using established measurement scales from prior studies, as detailed in the Measures section.

**Table 1:** Descriptive distribution of the sample.

Gender	Frequency (%)	Age	Frequency (%)
Male	87 (41.63)	18–24	42 (20.10)
Female	118 (56.46)	25–34	73 (34.93)
Prefer not to say	4 (1.91)	35–44	63 (30.14)
		45 and above	31 (14.83)
**Nationality**	**Frequency (%)**	**Education level**	**Frequency (%)**
United States	80 (38.28)	Bachelor's degree	110 (52.63)
China	45 (21.53)	Master's degree	80 (38.28)
Other countries	84 (40.19)	Doctoral degree	19 (9.09)

Eligibility was established through a screening question before the main questionnaire: “Have you seen or followed any virtual influencer or virtual activist on social media within the past 6 months?” Only respondents who answered affirmatively were permitted to proceed. Data quality was maintained through Prolific's participant monitoring procedures, as well as our own attention checks, screening criteria, and response-quality assessment.

In total, 299 responses were collected. The exclusion process was conducted before the final analysis. Responses were removed if participants failed the screening question, which required prior exposure to or familiarity with virtual influencers or virtual activists, did not provide the required Prolific completion code after completing the questionnaire, or exceeded the expected completion time and were screened out. After applying these exclusion criteria, 209 valid responses remained for analysis. The median completion time recorded by Prolific was approximately 7 min and 10 s. Among these respondents, 41.63 percent identified as male and 34.93 percent of them are aged between 25 and 34 years old. Table 1 summarizes the sample characteristics.

### 3.2 Measures

The questionnaire contained 26 scale items, excluding the screening question and demographic questions. This study utilized a seven-point Likert scale for survey responses, ranging from 1 (strongly disagree) to 7 (strongly agree). To evaluate anthropomorphism, scales proposed by Golossenko et al. (2020) were adapted. There are four key dimensions: appearance, moral virtue, cognitive experience, and conscious emotional, each measured using three items. Attitudes toward the influencer were assessed using three items adapted from Chetioui et al. (2020). Similarly, attitudes toward environmental self-identity were gauged employing three items based on the work of van der Werff et al. (2013). Parasocial interaction was measured with five items derived from Russell et al. (2006). Lastly, the propensity to engage in prosocial behaviors was evaluated using a scale adapted from Akerlof and Kranton (2002) and Shin and Kim (2018).

### 3.3 Analytical methods

This study employed a sequential multi-method approach by combining partial least squares structural modeling (PLS-SEM) and fuzzy set qualitative comparative analysis (fsQCA) for data analysis. First, the theoretical model was assessed using SmartPLS v4.0, as PLS-SEM is suited for the development and validation of explanatory theoretical models (Hair et al., 2017). While PLS-SEM offers strong advantages in handling complex models (Hair et al., 2017), it is limited in its ability to capture equality and causal asymmetry, especially in how different combinations of variables determine the outcomes.

To address these limitations, fsQCA was applied in the second stage of analysis, following the recommendations of Fiss (2011). fsQCA allows for the identification of multiple causal pathways and provides more insights into complex models by considering causal asymmetric relationships (Fang et al., 2016). Recent research increasingly applies fsQCA to complement and extend PLS-SEM results, thereby offering a more holistic perspective (Kang et al., 2024; Sobgo et al., 2025).

## 4 Analysis and results

### 4.1 Measurement model

In this study, a two-stage approach was employed to evaluate the measurement model. In the first stage, lower-order constructs were assessed prior to the higher-order construct. Consistent with Golossenko et al. (2020), anthropomorphism was conceptualized as a multidimensional construct comprising four dimensions: appeal, moral value, cognitive experience, and conscious emotion. Following Hair et al. (2017), reliability, convergent validity, and discriminant validity were examined to assess the measurement model. As shown in Table 2, Cronbach's alpha and composite reliability values exceeded the recommended threshold of 0.70, while the average variance extracted (AVE) for each construct surpassed the 0.50 threshold. Based on Table 2, discriminant validity was further assessed using heterotrait-monotrait ratio (HTMT), with all values falling below the suggested 0.90 threshold, thereby confirming the absence of discriminant validity issues.

**Table 2:** Measurement model.

Construct	Item	Loading	Cronbach's Alpha	Composite reliability	Average variance extracted
Appeal	A1: This virtual influencer looks human-like	0.952	0.939	0.961	0.892
	A2: This virtual influencer looks life-like	0.937			
	A3: This virtual influencer has a human-like appearance	0.944			
Moral value	MV1: This virtual influencer is trustworthy	0.960	0.955	0.971	0.918
	MV2: This virtual influencer is honest	0.961			
	MV3: This virtual influencer is principled	0.954			
Cognitive experience	CE1: This virtual influencer can engage in a great deal of thought	0.932	0.969	0.980	0.942
	CE2: This virtual influencer can imagine things on its own	0.948			
	CE3: This virtual influencer is capable of reasoning	0.941			
Conscious emotional	COE1: This virtual influencer can experience remorse over the actions which it deems to be shameful	0.972	0.934	0.958	0.884
	COE2: This virtual influencer can experience guilt when it hurts someone with its behavior	0.965			
	COE3: This virtual can experience shame when people have negative views and judgements about it	0.976			
Parasocial interaction	PI1: This virtual influencer seems to understand the things I want to know	0.840	0.931	0.948	0.784
	PI2: I would like to meet this virtual influencer in person	0.872			
	PI3: When I see a post of this virtual influencer, I feel as if I am part of the group	0.926			
	PI4: This virtual influencer feels like an old friend	0.890			
	PI5: I like to compare my ideas with what this virtual influencer says	0.897			
Attitude toward influencer	ATT1: I do believe that this activist influencer serves as a model for me.	0.892	0.885	0.929	0.813
	ATT2: I do believe that this activist influencer presents interesting content.	0.878			
	ATT3: I do consider this activist influencer as a reliable source of information and discovery	0.934			
Environmental self-identity	ESI1: Acting environmentally friendly is an important part of who I am	0.919	0.927	0.954	0.873
	ESI2: I am the type of person who acts environmentally friendly	0.938			
	ESI3: I see myself as an environmentally friendly person	0.945			
Intention to engage in prosocial behaviors	INT1: Since seeing this activist influencer's post, I want to save the environment through my efforts.	0.941	0.950	0.968	0.910
	INT2: Since seeing this activist influencer's post, I want to participate in various environmental activities to save the environment.	0.969			
	INT3: Since seeing this activist influencer's post, I want to do something more meaningful for others and society.	0.951			

In the second stage, the higher-order formative construct was evaluated for potential collinearity issues through variance inflation factor (VIF) values. Following the recommendation of Hair Jr et al. (2021), VIF values below 5 indicate no multicollinearity concerns. Results in Table 3 demonstrate that all anthropomorphism dimensions met this criterion, confirming that collinearity was not an issue.

**Table 3:** Heterotrait-monotrait ratio (HTMT) matrix.

Construct	1	2	3	4	5	6	7	8
1. Appeal								
2. Attitude toward influencer	0.547							
3. Cognitive experience	0.486	0.763						
4. Conscious emotional	0.327	0.577	0.780					
5. Environmental self-identity	0.335	0.517	0.325	0.222				
6. Intention to engage in prosocial behaviors	0.502	0.837	0.619	0.468	0.598			
7. Moral value	0.502	0.855	0.777	0.575	0.405	0.758		
8. Parasocial interaction	0.475	0.859	0.785	0.652	0.454	0.776	0.779	

Finally, the outer weights and loadings of the anthropomorphism were examined. As reported in Table 4, these values were statistically significant, thereby supporting the validity of anthropomorphism as a higher-order formative construct (Hair et al., 2017).

**Table 4:** Higher-order construct validity.

Construct	Item	Outer weight	*T*-statistics	*p*-values	Outer loading	VIF
Anthropomorphism	Appeal	0.225	11.376	0.000	0.642	1.340
	Moral value	0.364	19.695	0.000	0.876	2.302
	Cognitive experience	0.341	26.673	0.000	0.918	3.460
	Conscious emotional	0.277	15.572	0.000	0.804	2.238

### 4.2 Structural model

As presented in Table 5, anthropomorphism shows a significant positive effect on both parasocial interaction (β = 0.790; *p* < 0.001) and attitude toward influencer (β = 0.442; *p* < 0.001), thereby supporting H1 and H2. Furthermore, parasocial interaction demonstrated significant positive effects on attitude toward influencer (β = 0.435; *p* < 0.001) and environmental self-identity (β = 0.424; *p* < 0.001), supporting H4 and H5. Finally, both attitude toward influencer (β = 0.424; *p* < 0.001) and environmental self-identity (β = 0.240; *p* < 0.001) positively influenced intention to engage in prosocial behavior, thus confirming H6 and H7.

**Table 5:** Direct effects assessment.

Relationship	Path coefficient	STD error	*t*-value	*p*-value	5.0%	95.0%	*f* ^2^	Remark
H1: Anthropomorphism → Parasocial interaction	0.790	0.029	27.213	0.000	0.734	0.831	1.664	Supported
H2: Anthropomorphism → Attitude toward influencer	0.442	0.072	6.106	0.000	0.318	0.559	0.236	Supported
H3: Parasocial interaction → Attitude toward influencer	0.435	0.076	5.714	0.000	0.312	0.561	0.229	Supported
H4: Parasocial interaction → Environmental self-identity	0.424	0.061	6.923	0.000	0.315	0.517	0.219	Supported
H5: Parasocial interaction → Intention to engage in prosocial behavior	0.296	0.077	3.826	0.000	0.173	0.427	0.103	Supported
H6: Attitude toward influencer → Intention to engage in prosocial behavior	0.424	0.086	4.913	0.000	0.289	0.569	0.202	Supported
H7: Environmental self-identity → Intention to engage in prosocial behavior	0.240	0.061	3.947	0.000	0.141	0.340	0.137	Supported

As shown in Table 6, the results provide support for H8 and H9, indicating that anthropomorphism has an indirect effect on intention to engage in prosocial behavior through both attitude toward influencer and environmental self-identity. Following Hayes and Preacher (2010), the significance of these indirect effects was assessed using bias-corrected confidence intervals. The analysis revealed a significant and positive total effect of anthropomorphism on intention to engage in prosocial behavior (β = 0.286, *p* < 0.001). The direct effect remained significant (β = 0.296, *p* < 0.001), while the indirect effects through attitude toward the influencer (β = 0.185, *p* < 0.001, 97.5% CI [0.095, 0.311]) and environmental self-identity (β = 0.102, *p* < 0.001, 97.5% CI [0.050, 0.172]) confirmed the mediating roles of these constructs. Hair Jr et al. (2021) suggest that R2 values of 0.75, 0.5, and 0.25 represent significant, moderate, and limited explanatory power, respectively. The explanatory powers of attitude toward influencer, parasocial interaction, environmental self-identity, and intention to engage in prosocial behavior were 0.689, 0.625, 0.180, and 0.677, respectively.

**Table 6:** Mediation results.

Relationship	Total effect			Direct effect			Indirect effect						Remark
	Path coefficient	*t*-value	*p*-value	Path coefficient	*t*-value	*p*-value	Path coefficient	STD error	*t*-value	*p*-value	2.5%	97.5%	
H8: Parasocial interaction → Attitude toward influencer → Intention to engage in prosocial behaviors	0.286	5.510	0.000	0.296	3.826	0.000	0.185	0.055	3.362	0.001	0.095	0.311	Supported
H9: Parasocial interaction → Environmental self-identity → Intention to engage in prosocial behaviors	0.286	5.510	0.000	0.296	3.826	0.000	0.102	0.031	3.332	0.001	0.050	0.172	Supported

### 4.3 fsQCA analysis

In our second stage of analysis, fsQCA was employed to explore the data further and identify the combinations of antecedent conditions associated with intention to engage in prosocial behavior. As shown in Table 3 (HTMT), only a limited number of correlation coefficients exceeded the value of >0.60, suggesting that the relationship between anthropomorphism and intention to engage in prosocial behavior may be asymmetric. This indicates that different combinations of causal conditions can produce different outcomes. Therefore, using the results from PLS-SEM, fsQCA was applied to uncover alternative configuration paths that explain high levels of intention to engage in prosocial behaviors.

#### 4.3.1 Calibration

The first step involved calibrating the data into fuzzy set membership scores ranging from 0.0 to 1.0, where a score of 0.0 denotes full non-membership, 1.0 denotes full membership, and 0.5 represents the cross-over point, reflecting maximum ambiguity in set membership (Ragin, 2008). Data calibration was conducted using fsQCA v4.1. Following the approach suggested by Gligor and Bozkurt (2020), we applied the quartile method with thresholds specified at the 25% quantile for full non-membership, the 50% quantile for the cross-over point, and the 75% quantile for full membership.

#### 4.3.2 Necessary conditions analysis

The analysis of necessary conditions assessed whether the presence or absence of four preconditions (anthropomorphism, parasocial interaction, and environmental self-identity) is required for intention to engage in prosocial behavior. According to Yadav et al. (2024), a condition is classified as necessary when consistency exceeds 0.90 and coverage surpasses 0.50.

As shown in Table 7, the analysis of necessary conditions indicates that among four preconditions, only environmental self-identity is classified as a necessary condition for high intention to engage in prosocial behavior, with its consistency (0.984) and coverage (0.992). In contrast, anthropomorphism, parasocial interaction, and attitude toward the influencer fell below the threshold, indicating the absence of necessary conditions for either high or low intention to engage in prosocial behavior.

**Table 7:** Necessary conditions analysis for predicting intention to engage in prosocial behavior.

Causal condition	High intention		Low intention	
	Consistency	Coverage	Consistency	Coverage
Anthropomorphism	0.257	1.000	0.932	0.313
Parasocial interaction	0.774	0.999	0.925	0.103
Environmental self-identity	0.984	0.992	1.000	0.087
Attitude toward influencer	0.573	1.000	0.909	0.137

#### 4.3.3 Sufficient conditions analysis

Truth table analysis was used to determine solution configurations for high and low intention to engage in prosocial behavior. As shown in Table 8, two pathways were identified for high intention and three pathways for low intention. For high intention, both solutions indicate strong robustness, with consistency values of 0.996 and 1.000. The overall coverage is 0.888, indicating that nearly 89% of cases are explained. Between these two solutions, environmental self-identity emerges as a core condition, highlighting its central role in driving the intention.

**Table 8:** Solution configuration for achieving high and low PI.

Causal condition	Solution configurations				
	High		Low (~)		
	1	2	1	2	3
Situational factors					
Anthropomorphism	⊗			•	•
Parasocial interaction		•		⊗	
Environmental self-identity	•	•	⊗		
Attitude toward influencer		•			⊗
Raw coverage	0.820	0.559	0.906	0.930	0.932
Unique coverage	0.329	0.069	0.028	0.000	0.001
Consistency	0.996	1.000	0.833	0.549	0.434
Overall coverage	0.888		0.960		
Overall consistency	0.996		0.426		

• indicate the presence of a condition, and ⊗ indicate its absence.

In contrast, low intention pathways indicate weaker empirical support, with an overall consistency of 0.426. The configurations involve the absence of environmental self-identity, parasocial interaction, and attitude toward the influencer. The consistency levels for these three pathways fall below the threshold of 0.85, indicating a clear asymmetry.

## 5 Discussions

This study contributes to the discussion of virtual influencers in environmental-related behavior by showing that their influence is not limited to commercial persuasion. The findings suggest that anthropomorphic virtual influencers can support prosocial environmental intention when they generate parasocial interaction, favorable attitudes, and environmental self-identity. Importantly, environmental self-identity emerged as central to high prosocial intention, indicating that virtual influencers may be most effective when their messages help followers view environmental action as part of who they are.

This study examined how anthropomorphic design features in virtual influencers shape intention to engage in prosocial environmental behavior through parasocial interaction, attitude toward the influencer, and environmental self-identity. The structural model shows that anthropomorphism has a strong positive effect on parasocial interaction and a moderate positive effect on attitude, indicating that humanlike design strengthens both relational engagement and source evaluation. This positions anthropomorphism as the distinctive starting point of the persuasion process in virtual influencer activism, because artificial agents must first be perceived as socially meaningful before they can generate relational, evaluative, or identity-based responses. Parasocial interaction predicts attitude and environmental self-identity, and all three organismic states contribute significantly to prosocial intention. Mediation tests confirm that parasocial interaction affects intention indirectly through both attitude and environmental self-identity, supporting a dual pathway structure. The configurational analysis complements these findings by identifying environmental self-identity as a necessary condition for high intention and by revealing multiple sufficient combinations of anthropomorphism, parasocial interaction, attitude, and identity that lead to high prosocial intention.

The findings can be further understood through SCT. In this study, anthropomorphism significantly predicted parasocial interaction, suggesting that humanlike design cues make virtual influencers more salient and socially meaningful as symbolic models. This finding supports the SCT view that model characteristics shape whether audiences attend to and engage with a modeled message. While SCT has traditionally been applied to celebrities, peers, and media characters, our findings suggest that virtual influencers can also activate social cognitive processes when they are perceived as humanlike and relationally engaging. The results also refine SCT by showing that synthetic modeling works through parallel evaluative and identity-based mechanisms. The fsQCA finding that environmental self-identity is central to high prosocial intention further highlights the importance of self-regulatory internalization in virtual influencer activism.

The results extend anthropomorphism research by supporting a multidimensional higher order conceptualization in the context of virtual influencer activism. Appearance, moral virtue, cognitive experience, and conscious emotional qualities jointly form a coherent anthropomorphism construct that predicts downstream social and cognitive responses, in line with Golossenko et al. (2020). This finding strengthens arguments that perceived human likeness is not limited to visual realism but also depends on perceived morality, intelligence, and emotional expressiveness, as suggested by recent work on anthropomorphic and AI enabled agents (Dabiran et al., 2024; Geiselmann et al., 2023; Yin et al., 2026). This is especially important in environmental activism, where a virtual influencer must appear not only visually humanlike but also ethically sincere, cognitively capable, and emotionally aligned with the cause. In this sense, anthropomorphism functions as more than a design attribute; it provides the basis through which a virtual activist becomes interpretable as a credible social model.

The positive links between anthropomorphism, parasocial interaction, and attitude are consistent with prior studies showing that humanlike virtual influencers elicit stronger parasocial bonds and more favorable evaluations (Stein et al., 2022; De Cicco et al., 2024; Yoo et al., 2024). However, most earlier research has focused on commercial outcomes such as purchase intention and engagement (Casaló et al., 2020; Filieri et al., 2022; Han and Balabanis, 2023). By demonstrating that anthropomorphism operates through parasocial interaction to influence both attitudes and environmental self-identity in an environmental activism context, the present study shows that the same design principles can support prosocial rather than strictly market oriented goals. The findings therefore clarify why anthropomorphism is a unique feature of virtual influencer activism: unlike human influencers, whose humanness is assumed, virtual influencers depend on constructed humanlike cues to achieve social presence, credibility, and persuasive relevance. Parasocial interaction remains an important mechanism, but it should be understood as one pathway through which anthropomorphism becomes psychologically consequential rather than as the sole explanatory focus of the model.

The central role of environmental self-identity aligns with research that identifies identity as a proximal driver of environmental behavior (van der Werff et al., 2013, 2014; Brick and Lai, 2018; Ajibade and Boateng, 2021; Udall et al., 2021). Here, identity not only predicts intention but emerges as a necessary condition for high intention in the configurational analysis. This result indicates that activist virtual influencers are most effective when they contribute to the reinforcement of an environmentally responsible self-concept, rather than merely shaping evaluations of the messenger.

This identity-centered finding can also be interpreted through communication scholarship on selective exposure and identity reinforcement. Rather than suggesting that virtual activist influencers directly create prosocial intentions among neutral audiences, the results indicate that their influence may be stronger when audiences are already receptive to environmental values and encounter content that affirms those values. In this sense, virtual activist influencers may function less as direct persuasion agents and more as symbolic communicators that make pre-existing environmental identities more visible, socially validated, and personally meaningful. Anthropomorphism supports this process by making the virtual activist easier to interpret as a social actor, while parasocial interaction helps translate identity-consistent messages into relationally meaningful engagement. This interpretation extends the discussion beyond psychology and marketing by showing that virtual influencer activism may also operate as a mediated process of environmental identity reinforcement.

The identification of multiple sufficient configurations shows that there is no single dominant recipe for high prosocial intention. Some followers may be motivated by strong parasocial bonds combined with identity alignment, whereas others may rely on a combination of anthropomorphism, attitude, and identity. This asymmetry supports calls for configurational thinking in marketing and social media research (Fiss, 2011; Fang et al., 2016; Pappas and Woodside, 2021) and highlights that net effects captured by variance based methods do not fully describe the complexity of persuasion processes involving virtual agents.

The positive effects of anthropomorphism observed here suggest that, within the range of human likeness represented by the stimuli, anthropomorphic cues are beneficial for virtual influencer activism. Research on the uncanny valley indicates that very high realism without perfect fidelity may evoke discomfort and reduce trust (Mori et al., 2012; Stein et al., 2022). Although the present model does not test curvilinear effects, the findings can be interpreted as lying in a region where anthropomorphism supports parasocial bonding and prosocial intention. Therefore, the implication is not that virtual activists should always be made as humanlike as possible, but that anthropomorphic cues should be carefully calibrated so that visual appeal, moral positioning, cognitive competence, and emotional expression remain consistent with the environmental cause. Future conceptual work should examine potential non-linear relationships between specific facets of anthropomorphism and prosocial outcomes and consider whether the optimal level of appearance realism and emotional intensity may vary across causes, cultures, and audience segments.

## 6 Conclusion, implications, and limitations

This study extends the anthropomorphism and prosocial intention paradigm by demonstrating that anthropomorphic cues not only enhance followers' perceptions but also activate cognitive and emotional processes that strengthen parasocial interaction and shape intention to engage in prosocial behavior.

Theoretically, the study formalizes activist anthropomorphism as a higher order construct composed of appearance, moral virtue, cognitive experience, and conscious emotional qualities, extending recent anthropomorphism work into an environmental activism setting and clarifying how these cues operate through parasocial interaction to influence both attitudes and environmental self identity. It also demonstrates that identity is not merely an auxiliary variable but a necessary condition for high prosocial intention in this context, reinforcing identity based accounts of environmental behavior. Methodologically, the research shows the usefulness of combining a second order formative specification of anthropomorphism with a mixed PLS SEM and fsQCA strategy, illustrating how symmetric and asymmetric analyses together yield a more complete picture of persuasion processes involving virtual agents.

For practitioners, the results indicate that designing effective virtual activists requires more than attractive avatar aesthetics. Brands, non-governmental organizations, and public agencies should develop virtual influencers who are visually humanlike, morally principled, cognitively competent, and emotionally engaged with environmental issues. Content should illustrate ethical decision making, provide clear and credible explanations of environmental problems, and express emotions that match the gravity of the cause. Campaigns should be structured to cultivate ongoing parasocial relationships through consistent storytelling, interaction, and character development, since parasocial interaction is a key mechanism through which anthropomorphism shapes both attitudes and identity.

The central role of environmental self-identity suggests that campaigns should support identity construction by framing behaviors and calls to action in terms of what it means to be an environmentally responsible person. Virtual activists can invite followers to see sustainable choices as expressions of who they are, rather than isolated actions. At a societal level, anthropomorphically designed virtual influencers can serve as scalable advocates who repeatedly model pro environmental norms in ways that are tailored to different audiences.

At the same time, the increasing anthropomorphisation of artificial advocates raises ethical concerns that should not be overlooked. Because highly humanlike virtual activists may create feelings of familiarity, trust, or emotional attachment, designers should avoid using anthropomorphic cues in ways that obscure the artificial and strategically managed nature of these agents. Transparency about the virtual influencer's non-human identity, institutional sponsor, and advocacy purpose is therefore important for reducing risks of manipulation and preserving audience autonomy. These concerns are especially relevant in public advocacy, where artificial communicators may gradually supplement or replace human advocates. Rather than maximizing human likeness uncritically, practitioners should pursue calibrated and transparent anthropomorphism that supports engagement while maintaining authenticity, accountability, and ethical boundaries.

Our data were collected from Prolific respondents familiar with virtual influencer activism using purposive sampling. While appropriate for recruiting participants with relevant prior exposure, this approach limits generalisability to broader or nationally representative populations, particularly those unfamiliar with virtual influencers or exposed to environmental advocacy through other media channels. Future studies could employ cross-cultural or nationally representative samples to examine whether the effects of anthropomorphism, parasocial interaction, and environmental self-identity vary across different socio-cultural settings. Future research could also examine whether the institutional source behind a virtual activist influencer shapes audience responses. For example, virtual activists created or sponsored by governments, non-governmental organizations, private companies, or independent advocacy groups may generate different levels of trust, credibility, and persuasive acceptance. This would clarify whether the effectiveness of virtual activist influencers depends not only on anthropomorphic design, but also on the perceived legitimacy of the institution behind them. This study employed a cross-sectional survey design, which restricts causal inference. Although PLS-SEM and fsQCA provided robust evidence, longitudinal or experimental designs would allow for stronger causal claims. Future research could examine how exposure to anthropomorphic cues over time shapes parasocial relationships and environmental self-identity, and whether these factors drive sustained prosocial behavior.

## Data Availability

The raw data supporting the conclusions of this article will be made available by the authors, without undue reservation.
